# Evaluating the Readability and Understandability of Online Patient Educational Material for Percutaneous Coronary Intervention in Canada

**DOI:** 10.1016/j.cjco.2024.11.012

**Published:** 2024-11-25

**Authors:** Raumil V. Patel, Denis Qeska, Jennifer M. Amadio, Nicolas Bowers, Andrew C.T. Ha, Harindra C. Wijeysundera

**Affiliations:** a Temerty Faculty of Medicine, University of Toronto, Toronto, Ontario, Canada; b Division of Cardiology, Department of Medicine, Schulich Heart Program, Sunnybrook Health Sciences Centre, Toronto, Ontario, Canada; c Division of Cardiology, Department of Medicine, Peter Munk Cardiac Centre, Toronto General Hospital, University Health Network, Toronto, Ontario, Canada; d Institute for Health Policy, Management, and Evaluation, Toronto, Ontario, Canada; e Institute for Clinical Evaluative Sciences, Toronto, Ontario, Canada; f Sunnybrook Research Institute, Toronto, Ontario, Canada

## Abstract

**Background:**

Percutaneous coronary intervention (PCI) is the most common treatment for coronary artery disease revascularization. Many patients undergoing PCI may seek educational information online, but the reliability of such resources remains uncertain. This study seeks to assess the readability and understandability of online patient resources for PCI from Canadian hospital sources.

**Methods:**

We performed a descriptive study evaluating online educational materials pertaining to PCI hosted by all Canadian hospitals that perform the procedure. The primary outcomes were readability, assessed using the Flesch-Kincaid Grade Level (FKGL) and Scolarius score, and understandability plus actionability, as assessed using the Patient Education Materials Assessment Tool (PEMAT). Educational clinical material is recommended to be written at an FKGL between 6 and 8. A score between 50 and 89 on the Scolarius tool suggests the text is readable by most adults, and a PEMAT score >70% corresponds to an understandable and actionable educational material.

**Results:**

A total of 29 Canadian hospitals performing PCI and hosting unique educational content were identified. Only 71% of PCI-capable hospitals provide relevant online educational resources to patients. The average FKGL of the analyzed content was 10 (range 5-18) and the average Scolarius score was 127.8 (range 79-173). The average total PEMAT print score was 46.1%, whereas the average total PEMAT audiovisual score was 71.8%.

**Conclusions:**

Most of the educational material pertaining to PCI created by Canadian hospitals is in English and print format, and of poor readability, understandability, and actionability. Audiovisual materials perform better but are sparsely used.

## Graphical abstract

**Figure:**
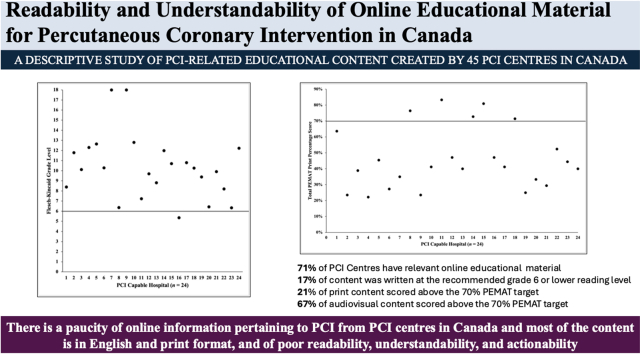

## Résumé

**Contexte:**

L’intervention coronarienne percutanée (ICP) est le traitement de revascularisation le plus fréquent des coronaropathies. De nombreux patients qui subissent une ICP peuvent chercher à s’informer en ligne sur le sujet, mais la fiabilité de telles ressources demeure incertaine. Cette étude vise à évaluer la lisibilité et l’intelligibilité des ressources en ligne qui portent sur l’ICP et qui sont mises à la disposition des patients par des hôpitaux canadiens.

**Méthodologie:**

Nous avons mené une étude descriptive visant à évaluer le matériel éducatif en ligne portant sur les ICP offert par tous les hôpitaux canadiens qui réalisent cette intervention. Les principaux critères d’évaluation étaient la lisibilité, évaluée par le score FKGL (*Flesch-Kincaid Grade Level*) et le score Scolarius, ainsi que l’intelligibilité et l’exploitabilité, évaluées à l’aide de l’outil PEMAT (*Patient Education Materials Assessment Tool*). Il est recommandé de rédiger le matériel d’éducation clinique en fonction d’un score FKGL se situant entre 6 et 8. Un score Scolarius entre 50 et 89 indique que le texte est lisible par la plupart des adultes, et un score PEMAT supérieur à 70 % correspond à du matériel éducatif intelligible et exploitable.

**Résultats:**

Au total, 29 hôpitaux canadiens réalisant des ICP et offrant du matériel éducatif unique ont été retenus. Seulement 71 % des hôpitaux pouvant réaliser des ICP fournissent aux patients des ressources éducatives en ligne pertinentes. Le score FKGL moyen du contenu analysé était de 10 (min.-max. : 5 - 18) et le score Scolarius moyen était de 127,8 (min.-max. : 79 - 173). Le score PEMAT total moyen pour les documents imprimés était de 46,1 %, alors qu’il était de 71,8 % pour les documents audiovisuels.

**Conclusions:**

La majeure partie du matériel éducatif portant sur les ICP créé par les hôpitaux canadiens est en anglais et en format imprimé et est difficilement lisible, intelligible et exploitable. Le matériel audiovisuel obtient de meilleurs résultats, mais il est rarement utilisé.

Cardiovascular disease remains a prominent cause of global morbidity and mortality, primarily manifesting as angina in the context of stable coronary artery disease. Percutaneous coronary intervention (PCI) is a frontline therapeutic modality for revascularization, offering relief from angina symptoms. In Canada, nearly 100,000 PCIs are conducted annually,^1^ with the United States reporting more than 600,000 procedures each year.^2^ Despite advancements in safety protocols, PCI remains a complex procedure associated with infrequent but catastrophic complications including vessel perforation, stroke, and death.

Recognizing the intricacies of PCI and its potential impact on patients, it is imperative to acknowledge the informational needs of patients offered PCI. Patients often seek supplementary educational materials beyond interactions with their health care team to enhance their comprehension of the intervention and its potential outcomes. With the widespread accessibility of online resources, patients increasingly turn to the Internet for health-related information.^3^^,^^4^ However, the reliability of online health content is variable, exhibiting disparities in accuracy, comprehensiveness, and readability. Studies reveal that a substantial proportion of online health information is presented at a reading level equivalent to grades 10 to 15 whereas the National Institute of Health recommends health materials be designed at a grade 6 to 7 reading level.^5^^,^^6^ Within Cardiology, the evaluation of online educational content for catheter ablation in atrial fibrillation found that a majority of websites provided information that was challenging to comprehend and lacked actionable insights.^7^ This underscores the pressing need to enhance the quality of online patient educational materials, facilitating improved understanding of diseases and therapeutic interventions. Moreover, this initiative seeks to empower patients in medical decision-making processes, fostering a sense of self-efficacy and enhancing health literacy, which is now identified as a crucial determinant of health.^8^^,^^9^

In light of these considerations, this article systematically compiles and assesses the quality of online patient resources on PCI originating from Canadian hospitals. This evaluation aims to provide insights into the current state of online patient educational materials, identify areas for improvement, and ultimately contribute to the development of patient-centric health care information.

## Material and Methods

We performed a descriptive study evaluating the quality of online patient educational material pertaining to PCI freely available to patients on Internet websites from Canadian hospitals performing PCI. The primary outcomes were readability assessed using the Flesch-Kincaid Grade Level (FKGL), Scolarius score, and understandability and actionability assessed using the Patient Education Materials Assessment Tool (PEMAT).

### Data sources and searches

The Canadian Institute for Health Information “Percutaneous Coronary Intervention (PCI) Volume by Province and Centre” indicator was used to identify all Canadian hospitals performing PCI. This indicator excludes Québec hospitals. Cardiologists practicing in Québec were contacted to identify all Québec hospitals performing PCI. The following search terms were entered on the Google search engine (www.google.ca) to locate the specific webpages or printable educational material on PCI for each hospital identified has created, “[Hospital name] + percutaneous coronary intervention OR PCI OR cardiac catheterization OR coronary angiogram OR angiographie coronarienne OR cathétérisme cardiaque.” If the hospital website had a built-in search function, the following terms were searched to identify other educational material relevant to PCI, “percutaneous coronary intervention OR PCI or cardiac catheterization or coronary angiogram OR angiographie coronarienne OR cathétérisme cardiaque.” Educational material on PCI either in text or audiovisual format were included for further analysis.

The data collection process was carried out from January 2024 to May 2024. Data were collected into a standard Microsoft Excel spreadsheet.

Two authors (R.P. and D.Q.) independently reviewed and evaluated the extracted educational material using the FKGL, Scolarius tool, and PEMAT. Inconsistences in evaluation were resolved through discussion until a consensus was reached.

### Flesch-Kincaid Grade Level

The FKGL is a formula that determines the approximate educational level a person will need to read and understand a particular text. The formula considers the average sentence length and the average syllables per word.^10^^,^^11^ The formula is validated and commonly used to assess the reading level of patient educational material. A high grade level corresponds to a more difficult to read and understand text. The highest possible grade level is 18, which corresponds to a college or university level education. The American Medical Association recommends that patient-focused health information be written at a grade 6 or lower reading level.^5^ Patient educational material presented in video format was excluded from this analysis.

### Scolarius tool

The Scolarius tool was created by Influence Communication and is freely available. It is designed to assess the readability of French content. It is based on multiple readability tests, and scores range from 50 to greater than 190. A score between 50 and 89 suggests the text is written at a primary level of education,^12^ which would be equivalent to the recommended grade 6 or lower reading level.

### PEMAT

The PEMAT assesses print and audiovisual educational material on understandability (which refers to the ability of patients to understand key messages) and actionability (which refers to the ability for patients to identify what actions they can take based on the information they read). The PEMAT was chosen as this tool has validity evidence with strong inter-rater reliability.^13^^,^^14^ Each domain (understandability and actionability) has several criterions used to evaluate the educational material. Applicable scores for each criterion include one (agree), zero (disagree), or NA (not applicable). By tallying the scores and dividing them by the maximum possible score and multiplying by 100, a total PEMAT score expressed as a percentage was obtained, ranging from zero to 100. A higher score corresponds to greater understandability and actionability. A PEMAT score at or below 70% corresponds to a poorly understandable or actionable educational material.^13^ Educational content in French was excluded from analysis with PEMAT as the tool has not been tested for the French language.

### Statistical analysis

Statistical analysis was performed using Microsoft Excel. Descriptive statistics were used to report the FKGL, Scolarius, and PEMAT scores.

## Results

**Figure 1:**
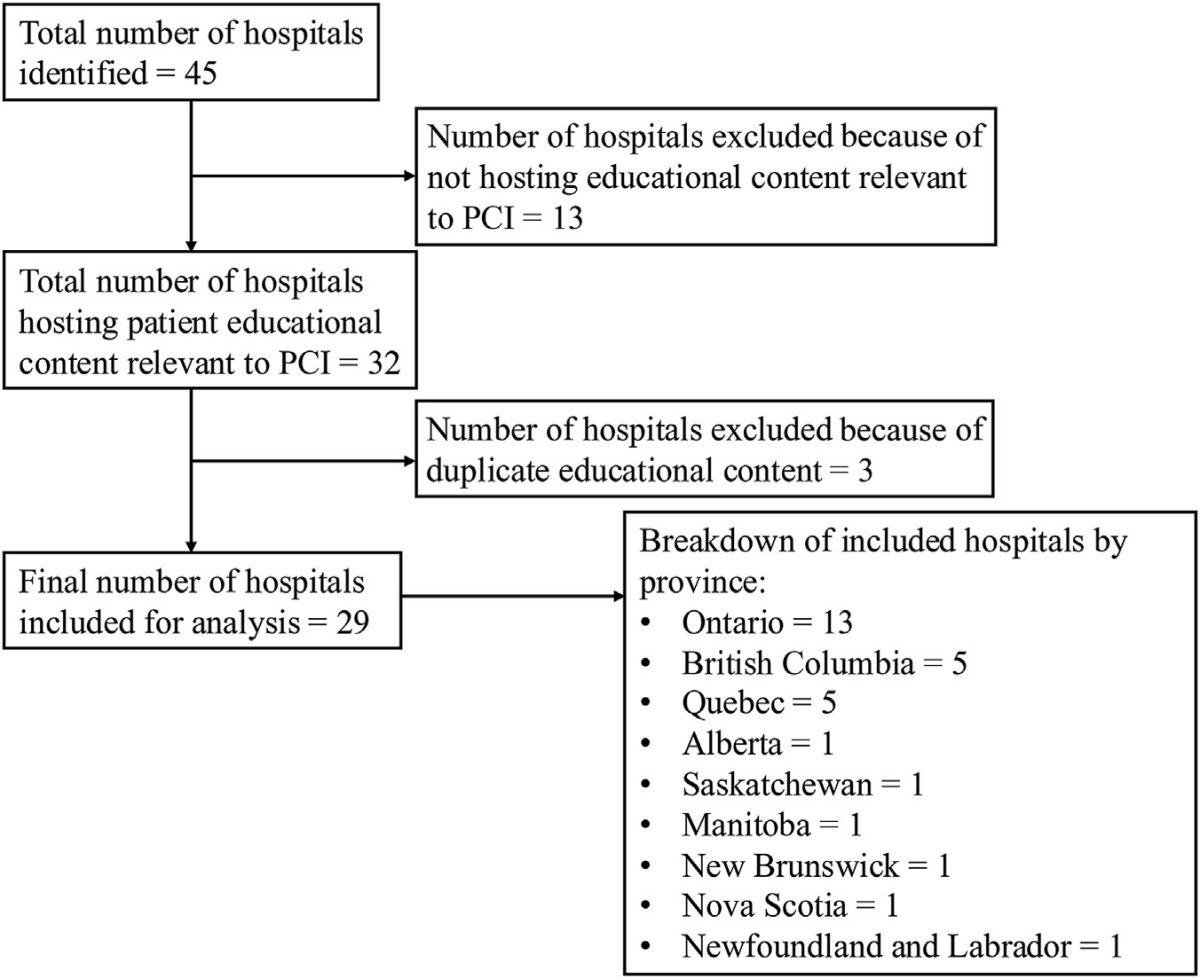
Flow diagram demonstrating inclusion and exclusion process for analyzed hospitals. PCI, percutaneous coronary intervention.

A total of 45 hospitals performing PCI were identified, 32 (71%) of which had created relevant online educational material for patients. Three hospitals were marked as duplicates and removed from analysis as they were from the same hospital system and shared the same educational content. A total of 29 hospitals were included in the final analysis (Fig. 1). Of the 29 hospitals, 13 were from Ontario, 5 from British Columbia, 5 from Québec, and 1 each from Alberta, Saskatchewan, Manitoba, New Brunswick, Nova Scotia, and Newfoundland and Labrador. All 5 Québec hospitals hosted educational content solely in French.

### Readability

**Figure 2:**
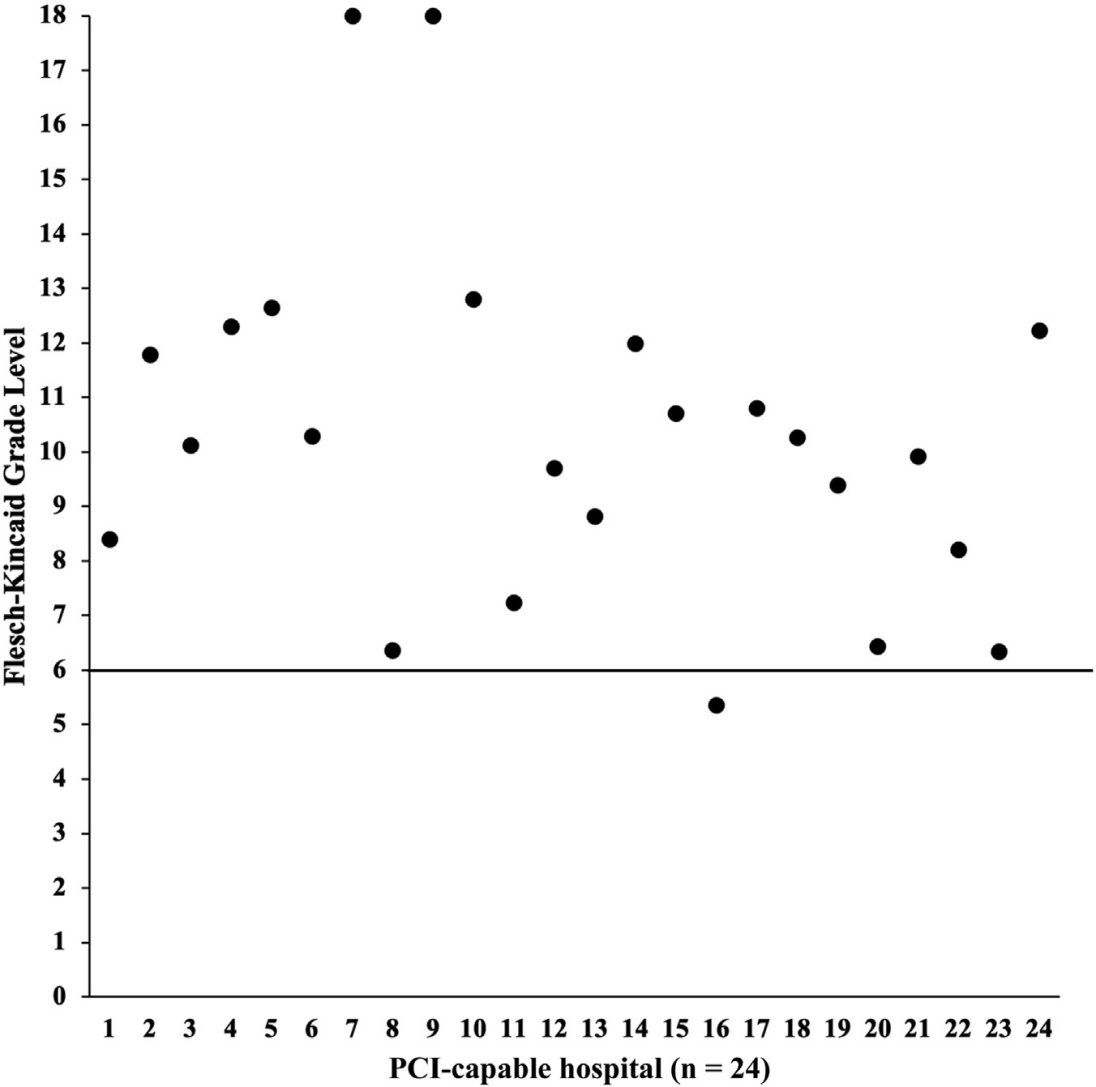
Scatter plot of the Flesch-Kincaid Grade Level for analyzed hospitals. Patient-focused text is recommended to be written at the grade 6 level. PCI, percutaneous coronary intervention.

**Figure 3:**
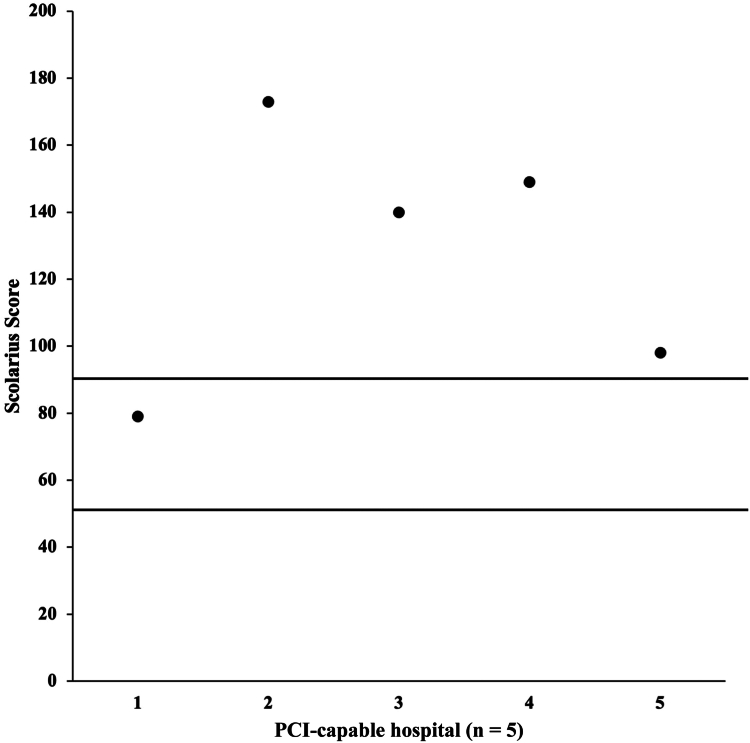
Scatter plot of the Scolarius score for analyzed hospitals. A score between 50 and 89 is ideal for patient focused text. PCI, percutaneous coronary intervention.

The average FKGL of the analyzed content was 10. The median was also 10. The highest grade level was 18, and the lowest grade level was 5. Only 4 of 24 websites (17%) were written at a grade level of 6 or lower (Fig. 2). The average Scolarius score was 127.8. The median was 140. The highest score was 173, and the lowest score was 79. Only 1 of the 5 websites (20%) scored within the ideal range of 50 to 89 (Fig. 3).

### Understandability and actionability

**Figure 4:**
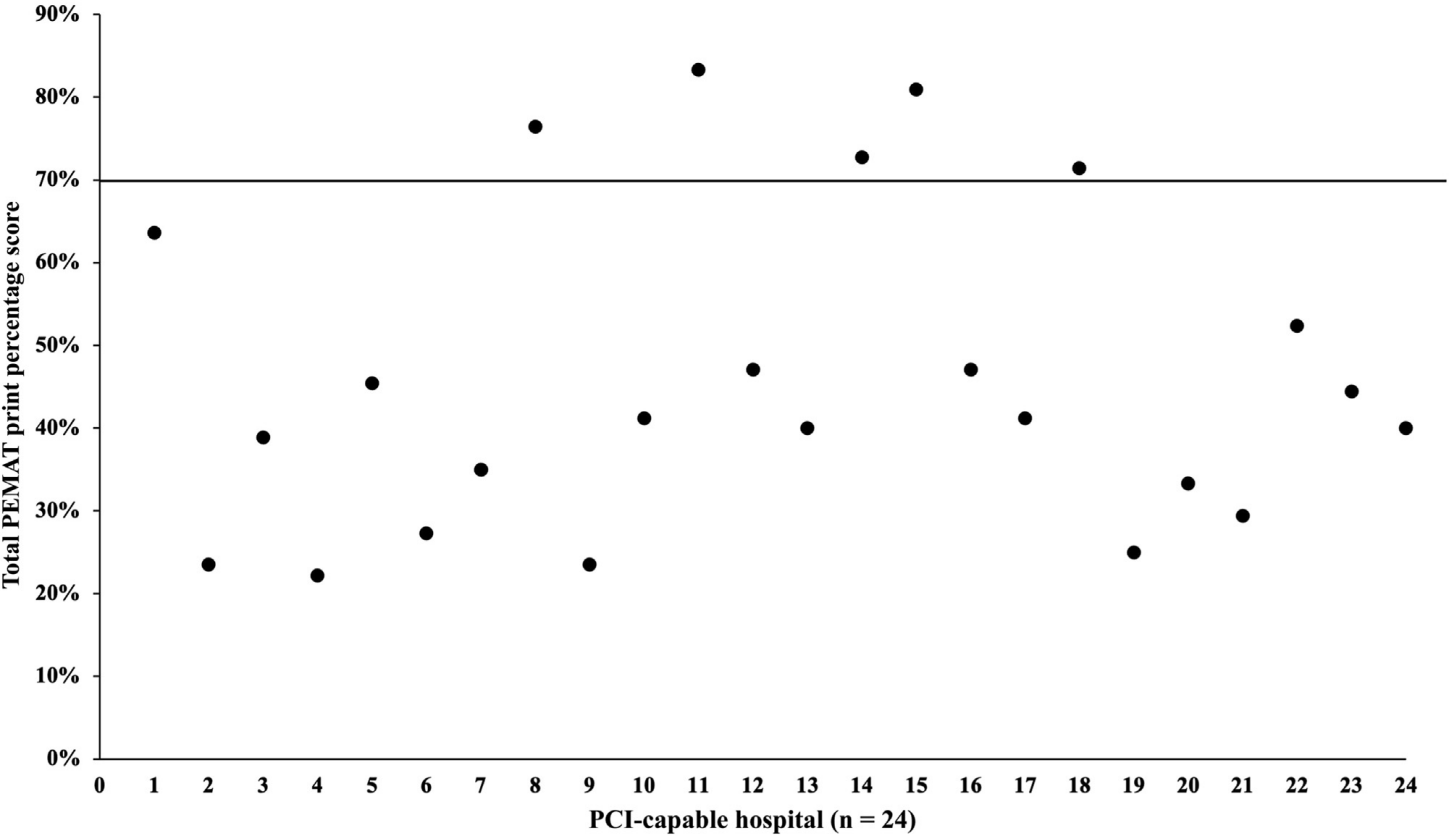
Scatter plot of the total percentage PEMAT print scores for analyzed hospitals. The target score is above 70%. PCI, percutaneous coronary intervention; PEMAT, Patient Education Materials Assessment Tool.

A PEMAT print score was calculated for all 24 hospitals with educational content in English language. The average total PEMAT print score for the analyzed content was 46.1%. The highest score was 83.3% and the lowest score was 22.2% (Fig. 4). Only 5 pieces (21%) of educational content scored higher than the target of 70%. The average total understandability and actionability scores were 54.3 and 24.3%, respectively.

Visual cues to highlight key information were included in 9 of 24 (37.5%) of the educational material. When considered more broadly, 12 of 24 (50%) of educational content included some form of visual aid (e.g., graph or illustration). The most frequently missed items were a summary (1/24) and lack of a tangible tool (1/24) or visual aid (0/24) to help patients take actionable steps.

**Figure 5:**
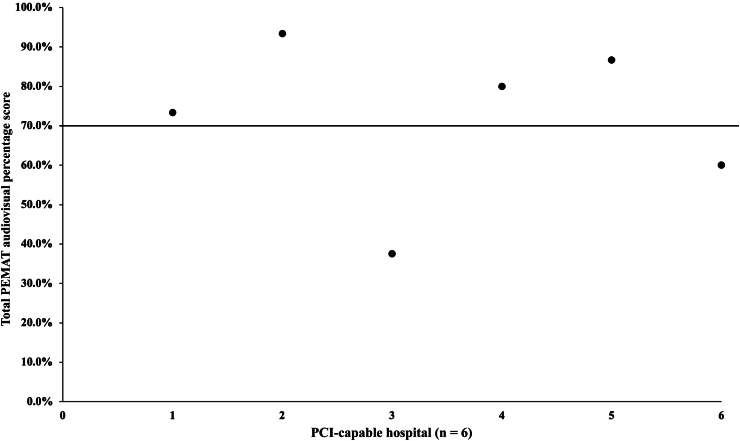
Scatter plot of the total percentage PEMAT audiovisual scores for analyzed hospitals. The target score is above 70%. PCI, percutaneous coronary intervention; PEMAT, Patient Education Materials Assessment Tool.

A total of 6 of the 24 hospitals (25%) studied provided educational content in audiovisual format. The average total PEMAT audiovisual score for the analyzed content was 71.8%. The highest score was 93.3%, and the lowest score was 37.5% (Fig. 5). Four pieces (67%) of educational content scored higher than the target of 70%. The average total understandability and actionability scores were 73.0% and 66.7% respectively. The most frequently missed items were the inclusion of informative headers (1/6) and a material summary (1/6).

## Discussion

The objective of our study was to systematically analyze the educational resources pertaining to PCI created by hospitals in Canada for readability, understandability, and actionability. We found that only 71% of PCI-capable hospitals in Canada provide relevant online patient education resources, and most of the content is written in English and available in print format with sparse use of audiovisual content. Only 17% of the analyzed content was written at an appropriate reading level, and similarly, only 21% of the print resources scored above target on the PEMAT scale for understandability and actionability. In contrast, audiovisual materials performed better, with 67% scoring above target on the PEMAT scale.

Our results suggest there is a scarcity of relevant online information made available to patients from PCI centres. This is problematic considering prior research has highlighted unmet educational needs among patients undergoing PCI during hospitalization and discharge,^15^^,^^16^ possibly prompting some to seek supplementary information online. Given the procedural complexity and increasing efficiency with a growing emphasis on expedited recovery pathways and same-day discharges post-PCI,^17^ the window for comprehensive patient education during the hospitalization is likely constrained. Moreover, urgency, such as in cases of myocardial infarction, may further limit the opportunity for in-depth discussions regarding procedural intricacies and postprocedure care. Altogether, this further supports the need for easily accessible online information relevant to PCI.

The high readability scores indicate potential comprehension challenges for a significant number of patients, especially in the context of a Canadian population where a significant proportion have a native language that is not English or French, and nearly 49% of Canadians possessing below-average literacy proficiency.^18^^,^^19^ This underscores the importance of health information being written at a sixth grade or lower reading level to mitigate disparities in health literacy.^5^^,^^8^^,^^9^ In addition, subpar understandability and actionability scores may further hinder patients’ ability to extract key information. Our findings suggest that most of the educational content analyzed lacked important components essential for mitigating readability issues, such as summary statements and the integration of visual cues and aids. This finding is consistent with prior work in this domain, which similarly uncovered deficiencies in online health information regarding catheter ablation for atrial fibrillation.^7^ We have built on this work by also analyzing audiovisual content. Despite the small sample size, our findings reveal notably higher total PEMAT scores, alongside improved understandability and actionability with audiovisual content compared to print content. This suggests that multimedia formats may be a more effective means of conveying complex medical information to patients by possibly overcoming language barriers and disabilities that impede reading ability.^20^

A review of our results also did not identify any factors (such as academic or community, PCI volume, or centre experience) associated with better readability or PEMAT scores. Nonetheless, we believe there are numerous changes health organizations can make to improve the quality of their online health information such as simplifying complex medical terminology and concepts, incorporating visual cues and aids, and if feasible, supplementing their print content with audiovisual material. However, the backbone to these changes should be patient partnership. The incorporation of end-users in the creation and development process is fundamental to ensuring that the information meets the diverse needs and preferences of patients.

Several limitations of our study warrant consideration. First, all content was evaluated by medical professionals and not end-users (the patients). Therefore, given discrepancies in health literacy between patients and professionals, our results may represent an upper estimate of user accessibility. Second, although the PEMAT tool facilitated assessment of the understandability and actionability of content, it did not encompass comprehensiveness, which is integral to overall educational material quality. Thus, material performing well on the PEMAT might have overlooked crucial educational components, such as the importance of strict adherence to antiplatelet therapy post-stent placement, not captured in our analysis. Third, online material represents just one facet of patient education, and the extent to which it influences decision-making compared with other methods remains unclear. Fourth, although we resolved discrepancies in rating through consensus, we did not use already-rated pre-existing materials to check internal validity of the tools used. Fifth, as the PEMAT tool has not been validated for use with the French language, we were only able to analyze French content for ease of readability. Lastly, our analysis focused solely on hospital websites, potentially overlooking third-party platforms hosting higher-quality educational material that may be more accessible to patients.

## Conclusions

In summary, not all PCI-capable Canadian hospitals provide online educational material pertaining to PCI and most of the content is in English and print format, and of poor readability, understandability, and actionability. Audiovisual material performs better but is sparsely used. As patients increasingly turn to the Internet for online health information, health care systems will need to revisit existing online health material to ensure it is available in an accessible and digestible manner that empowers patient decision making.
